# Maintenance of treatment gains up to 12-months following a three-week cognitive processing therapy-based intensive PTSD treatment programme for veterans

**DOI:** 10.1080/20008198.2020.1789324

**Published:** 2020-08-12

**Authors:** Philip Held, Alyson K. Zalta, Dale L. Smith, Jenna M. Bagley, Victoria L. Steigerwald, Randy A. Boley, Michelle Miller, Michael B. Brennan, Rebecca Van Horn, Mark H. Pollack

**Affiliations:** a Department of Psychiatry, Rush University Medical Center, Chicago, IL, USA; b Department of Psychological Science, University of California, Irvine, CA, USA; c Department of Behavioral Sciences, Olivet Nazarene University, Bourbonnais, IL, USA

**Keywords:** PTSD, intensive treatment, veterans, follow-up, posttraumatic cognitions, PTSD, 强化治疗, 退伍军人, 随访, 创伤后认知, TEPT, tratamiento intensivo, veteranos, seguimiento, cogniciones postraumáticas, • Intensive treatment programmes (ITPs) deliver treatment over the course of 1-3 weeks and show promise for reducing PTSD and depression symptoms. • This study showed that symptom reductions achieved during an ITP can be maintained for up to 12 months after treatment completion

## Abstract

**Background:**

Intensive treatment programmes (ITPs) have shown promise for reducing PTSD and depression symptoms. It is still unknown whether treatment gains are maintained following completion.

**Objective:**

This study examined whether veterans were able to maintain treatment gains for up to 12 months after an ITP for PTSD and whether reductions in negative posttrauma cognitions predicted treatment gain maintenance.

**Methods:**

209 veterans (62.7% male, mean age = 40.86 years) completed a 3-week, CPT-based ITP for PTSD. Participants’ PTSD (PCL-5) and depression (PHQ-9) symptoms were assessed at pre-treatment, post-treatment, and at 3-, 6-, and 12-month follow-up timepoints.

**Results:**

Despite small symptom increases from post-treatment to 3-month follow-up, significant and clinically meaningful reductions in PTSD and depression symptoms were reported from intake to 12 months follow-up (averaging >18 points on the PCL-5 and >6 points on the PHQ-9; *d* = 1.28, and *d* = 1.18, respectively). Greater reductions in negative posttrauma cognitions during treatment were associated with lower PTSD (*p* <.001) and depression (*p* =.005) severity at follow-up. Most veterans who completed the aftercare survey followed treatment recommendations and reported seeing a mental health provider at 3-, 6-, and 12-months post-treatment. Aftercare treatment did not significantly predict whether veterans maintained treatment gains at follow-up.

**Conclusions:**

Overall maintenance of treatment gains long-term suggests veterans may be able to apply skills acquired during the ITP following treatment. These findings further support the feasibility and effectiveness of intensive, trauma-focused, evidence-based therapy delivery.

## Abstract (es)

**Antecedentes**: Los programas de tratamiento intensivos (ITPs por sus siglas en inglés) han mostrado ser promisorios para reducir el TEPT y los síntomas depresivos. Se desconoce aún si las ganancias del tratamiento se mantienen después de la finalización. Este estudio examinó si los veteranos fueron capaces de mantener las ganancias del tratamiento después de 12 meses de un ITP para TEPT y si las reducciones de las cogniciones negativas postrauma predijeron la mantención de las ganancias del tratamiento.

**Método**: 209 veteranos (62,7% varones, edad media=40,86 años) completaron una ITP de 3 semana basado en CPT. Los síntomas de TEPT (PCL-5) y depresión (PHQ-9) de los participantes se evaluaron pre-tratamiento,post tratamiento y a los 3,6 y 12 meses de seguimiento.

**Resultados**: A pesar de un pequeño aumento de los síntomas a los tres meses de seguimiento después de terminado el tratamiento, se reportaron reducciones clínicamente significativas e importantes en el TEPT y síntomas depresivos desde el inicio hasta los 12 meses de seguimiento (un promedio ˃18 puntos en el PCL-5 y ˃6 puntos en el PHQ-9; *d*=1.28, y *d*=1.18, respectivamente. Las mayores reducciones en las cogniciones negativas postrauma durante el tratamiento se asociaron con una menor severidad del TEPT (*p*˂ .001) y depresión (*p*=.005) en el seguimiento. La mayoría de los veteranos que completaron la encuesta de cuidados posteriores siguieron las recomendaciones del tratamiento e informaron haber visto a algún profesional de salud mental a los 3, 6 y 12 meses post-tratamiento. Los cuidados posteriores al tratamiento no predijeron significativamente si los veteranos mantenían las ganancias del tratamiento en el seguimiento.

**Conclusiones**: el mantenimiento general de las ganancias del tratamiento a largo plazo sugiere que los veteranos pueden aplicar las habilidades adquiridas durante la PTI después del tratamiento.Estos hallazgos respaldan aún más la viabilidad y efectividad de la administración en forma intensiva de una terapia basada en la evidencia y centrada en el trauma.

## Abstract (zh)

**背景**:强化治疗计划 (ITP) 已展现出可减少PTSD和抑郁症状的希望。完成后是否能维持治疗获益仍是未知的。本研究考查了退伍军人是否能够在PTSD的ITP后维持长达12个月的治疗效果, 以及负性创伤后认知水平的降低是否可预测治疗效果的维持。

**方法**:209名退伍军人 (男性占62.7％, 平均年龄为 40.86岁) 完成了针对PTSD为期3周的基于CPT的ITP。在治疗前, 后以及3个月, 6个月和12个月的随访时间点评估参与者的PTSD (PCL-5) 和抑郁 (PHQ-9) 症状。

**结果**:尽管从治疗后到3个月的随访中症状有小幅增加, 但从治疗开始到随访12个月, PTSD和抑郁症状均报告有显著且具有临床意义的降低 (PCL-5平均> 18分, PHQ-9平均> 6分; 分别为d = 1.28和d = 1.18) 。治疗期间负性创伤后认知水平的更大降低与随访时更低的PTSD (p <.001) 和抑郁严重程度 (p =.005) 有关。大多数完成了后期护理调查的退伍军人都遵循治疗建议, 并报告在治疗后3个月, 6个月和12个月见过心理健康提供者。后期护理不能显著预测退伍军人在随访中是否维持治疗获益。

**结论**:长期维持治疗获益总体上表明, 退伍军人可能能够在治疗后运用ITP中获得的技能。这些发现进一步支持聚焦创伤的强化循证治疗的可行性和有效性。

Intensive treatment programmes (ITPs) are being used increasingly to address posttraumatic stress disorder (PTSD) (Held, Bagley, Klassen, & Pollack, 2019a). In ITPs, evidence-based treatments for PTSD, such as Cognitive Processing Therapy (CPT), Prolonged Exposure (PE), Cognitive Therapy for PTSD (CT-PTSD), and Eye Movement Desensitization and Reprocessing (EMDR) are condensed into one (Ehlers et al., 2014; Held et al., 2019b; Hendriks, Kleine, Broekman, Hendriks, & van Minnen, 2018; Méndez, Nijdam, June ter Heide, van der Aa, & Olff, 2018), two (Bryan et al., 2018; Harvey et al., 2017), or three (Zalta et al., 2018) weeks with daily sessions rather than traditional weekly psychotherapy (Held et al., 2019a). Although still relatively new, intensive treatment delivery formats have shown robust reductions in patients’ PTSD and depression symptoms in a matter of days (e.g., Beidel, Frueh, Neer, & Lejuez, 2017; Ehlers et al., 2014; Foa et al., 2018; Held et al., 2019b; Zalta et al., 2018). ITPs appear to produce similar or greater effect sizes compared to traditionally-delivered evidence-based treatments while utilizing a shortened timeframe and achieving treatment completion rates between 90–95% (Harvey et al., 2019).

Given the abbreviated duration, a critical question is whether patients are able to maintain their gains following completion of an ITP. Research examining symptom maintenance following intensive treatments is sparse but encouraging. Large effect sizes have been found for treatment gains maintained at 3- and 6-month follow-up (Beidel et al., 2017; Hendriks et al., 2018) and up to 40 weeks (Ehlers et al., 2014). It is also necessary to identify those individuals at risk for symptom worsening after an intensive PTSD treatment. Reductions in negative posttrauma cognitions (i.e., beliefs about the event, oneself, others, and the world) over the course of treatment, which are accepted as a well-known treatment mechanism for cognitive-behavioural interventions (Zalta, 2015), have been shown to predict longer-term symptom maintenance for up to 10 years following the completion of traditionally-delivered CPT (Resick et al., 2012; Resick, Nishith, Weaver, Astin, & Feuer, 2002). Changes in negative posttrauma cognitions appear to be equally as critical to achieve PTSD symptom change in ITPs as they are in traditionally-delivered formats (Zalta et al., 2018). Yet, no published study has examined this particular mechanism as a predictor of longer-term symptom maintenance following ITP completion. Additionally, no published research has examined other factors, such as baseline or post-treatment symptom severity, symptom changes during treatment, or demographic factors as predictors of symptom maintenance following ITPs.

The purpose of the present study was to address the highlighted gaps in the current literature associated with longer-term outcomes following ITP completion. Specifically, the goals were to 1) determine whether patients who completed a 3-week CPT-based intensive PTSD treatment program were able to maintain their treatment gains for up to 12-months, and 2) examine reductions in negative posttrauma cognitions over the course of the ITP as a predictor of PTSD and depression symptom maintenance up to 12 months after program completion while also 3) exploring age, sex, symptom severity at baseline and post-treatment, and changes in symptom severity over the course of treatment as predictors of PTSD and depression symptom worsening following ITP completion. Based on the findings from traditionally-delivered PTSD treatments and intensive PTSD treatments highlighted above, we hypothesized that PTSD and depression symptoms would be maintained for up to 12 months and that reductions in negative posttrauma cognitions over the course of treatment would predict reduced PTSD and depression severity during the follow-up time period. Given the limited amount of research in this area, we did not have specific directional hypotheses related to the other predictors of symptom worsening.

## 1. Methods

### 1.1. Programme description

Data for this case series study was drawn from a 3-week CPT-based ITP for service members and veterans (hereafter collectively referred to as veterans). The present sample substantially overlaps with published data examining programme outcomes from pre- to post-treatment (Zalta et al., 2018) but differs as the present study examines 3-, 6-, and 12-month follow-up timepoints. The intensive programme is housed within a non-VA mental health clinic at Rush University Medical Centre in the USA and provides mental health services at no cost to veterans and their families, regardless of military discharge characterization or service status. As part of the 3-week ITP, veterans were offered 14 daily 50-minute sessions of individual CPT, 13 daily 120-minute sessions of group CPT, 13 daily 75-minute group sessions of an adapted version of Mindfulness Based Stress Reduction (Kabat-Zinn, 1990), and 13 daily 50-minute group sessions of trauma-sensitive yoga, as well as daily psychoeducation. As detailed elsewhere (Held, Klassen, & Boley et al., 2019c), on average veterans participated in 13/14 sessions of individual CPT (mode = 14), 12/13 sessions of group CPT (mode = 13), 12/13 group sessions of mindfulness (mode = 13), and 10/12 group sessions of yoga (mode = 10) over the course of the 14 days of intensive PTSD treatment. The different types of interventions were combined to offer a holistic treatment approach and to help the individuals acquire a range of skills to manage both their posttraumatic stress symptoms and general life stress following the completion of the programme.

Veterans were eligible if they had a current primary diagnosis of PTSD based on the Clinician Administered PTSD Scale for DSM-5 (CAPS-5) (Weathers et al., 2018). Exclusion criteria included active suicidality or homicidality (i.e., plan and/or attempt within the past 60 days), current engagement in significant non-suicidal self-harm, active mania or psychosis, active eating disorders, and/or active substance use that would interfere with an individual’s ability to participate in the ITP or pose a risk of physiological withdrawal possibly requiring medical attention. The programme offered two treatment tracks focused on military sexual trauma or combat-related trauma. Individuals were assigned to the treatment tracks based on the index trauma identified on the CAPS-5. The programming of the two cohort types was nearly identical (Zalta et al., 2018). Following treatment, all veterans, regardless of their post-treatment symptom severity, received referrals for continued mental health care in their home area. Veterans were encouraged to meet with an individual psychotherapist in their home environment within two weeks of completing the ITP and follow the treatment plan recommended by their home provider. For a more detailed description of the programme, including acceptance and patient flow, as well as pre- to post-treatment outcomes, see Held et al. (2019c) and Zalta et al. (2018), respectively.

## 2. Participants

The sample for the present study consisted of 209 veterans (92.8% discharged, retired, or medically retired; 7.2% Active Duty, Reserve, National Guard, or Inactive Ready Reserve) who completed the 3-week ITP between April 2016 and March 2018 (see Table 1). The range was limited to these dates to ensure that all veterans in the present sample had reached the 12-month follow-up timepoint. On average, veterans were 40.86 years old at the start of treatment (*SD* = 9.46, Range = 24–70 years). Most veterans in the sample were male (62.7%), served after 11 September 2001 (88.5%), were deployed at least once (80.9%), and were not local (89.0%; defined as living outside of a 60-mile radius of the clinic).

**Table 1.** Demographic characteristics.

Variable	*n*	%	[M (SD)]
Age	209		[40.86 (9.46)]
Sex			
Male	131	62.7	
Ethnicity			
Not Hispanic or Latino	168	80.4	
Race			
American Indian/Alaskan Native	6	2.9	
Asian	1	.5	
Black or African American	41	19.6	
Native Hawaiian/Pacific Islander	3	1.4	
Other	16	7.7	
White	142	67.9	
Marital Status			
Divorced	41	19.6	
Domestic Partner	1	.5	
Legally separated	17	8.1	
Married	109	52.2	
Single	38	18.2	
Widowed	3	1.4	
Military Service Branch			
Air Force	19	9.1	
Army	140	67.0	
Coast Guard	2	.9	
Marines	28	13.4	
Navy	20	9.6	
Military Pay Grade			
E1 – E3	24	11.5	
E4 – E9	173	82.8	
Officer	12	5.7	
Discharge Status			
Active Duty	7	3.3	
Discharged	142	68.0	
Inactive ready Reserve	2	1.0	
Medically retired	39	18.7	
National Guard	3	1.4	
Reserves	3	1.4	
Retired	13	6.2	
Discharge Characterization			
General	4	1.9	
Honourable	153	73.2	
Medical	44	21.1	
Not Applicable	7	3.3	
Other than Honourable Conditions	1	.5	
Service Era			
Post-9/11	185	88.5	
Deployed^a^			
Yes	169	80.9	

Total *N* = 209. ^a^ Deployed *n* = 208.

### 2.1. Assessment procedures

All study procedures were approved by the Institutional Review Board. A waiver of consent was obtained because all assessments were collected as part of routine clinical care procedures. Information on demographic and military characteristics was collected at intake using the electronic medical record. Clinical assessment data for the present study was collected electronically using Qualtrics, an internet-based survey software (Qualtrics, 2019). Clinical assessments were administered at pre-treatment (within 2-weeks prior to ITP start), post-treatment (final day of the programme), and at 3-, 6-, and 12-month follow-up periods. The assessments during the post-treatment and follow-up periods were limited to self-report measures in an attempt to reduce the burden placed on the veterans, who were asked to complete these evaluations as part of a clinical programme evaluation. Veterans were emailed a link to complete follow-up surveys. Programme staff called veterans who did not complete their follow-up surveys within one week of the time they were sent. A total of three attempts were made to reach an individual and encourage the completion of the follow-up surveys. Veterans were compensated 20 USD in the form of an electronic gift card for the completion of surveys at each follow-up timepoint.

### 2.2. Measures

#### 2.2.1. Demographic and military characteristics

During the intake evaluation, veterans reported age, sex, race, ethnicity, marital status, last or current military pay grade, discharge characterization, branch of service, service era, discharge status, and deployment history.

#### 2.2.2. Aftercare survey

At 3-, 6-, and 12-months following programme completion, veterans were surveyed to determine if they were currently seeing a behavioural health provider. The specific kind or frequency of the mental health care they were receiving was not captured in the aftercare survey.

#### 2.2.3. The PTSD checklist for DSM-5 (PCL-5)

The PCL-5 (Weathers et al., 2013) is a 20-item self-report measure of PTSD symptom severity over the past month based on DSM-5 diagnostic criteria (Bovin et al., 2016; Weathers et al., 2013; Wortmann et al., 2016). For post-treatment, participants rated their symptom severity over the past week since the programme was only 3 weeks long. Internal reliability for the PCL-5 ranged from .89-.96.

#### 2.2.4. Patient health questionnaire (PHQ-9)

The PHQ-9 (Kroenke, Spitzer, & Williams, 2001) is a 9-item self-report measure of depression symptoms occurring during the past two weeks. The measure was administered at intake, post-treatment, and all post-treatment follow-up time points. Internal reliability for the PHQ-9 in the present sample ranged from .80-.91.

#### 2.2.5. Posttraumatic cognitions inventory (PTCI)

The PTCI (Foa, Ehlers, Clark, Tolin, & Orsillo, 1999) is a 33-item self-report measure that assesses trauma-related cognitions including self-blame, negative cognitions about the self, and negative cognitions about others and the world (Sexton, Davis, Bennett, Morris, & Rauch, 2018) that was administered at intake and at post-treatment. Internal reliability for the PTCI in the present sample was .95 at intake and .98 at post-treatment.

### 2.3. Analyses

Descriptive analyses examining demographic characteristics of the sample, follow-up survey completion rates, and the proportion of veterans who reported seeing a mental health provider following programme completion were performed using SPSS 26 (SPSS, 2019). Random effects models were used to examine longitudinal trends in PCL-5 symptom severity across pre- and post-treatment, 3-, 6-, and 12-month follow-up, as well as timepoint contrasts. Quadratic and cubic time components were explored due to expectations that outcome changes over time may not be linear. To determine whether reductions in negative posttrauma cognitions from pre- to post-treatment would predict longer-term outcomes, change in PTCI scores from pre- to post-treatment was examined as a covariate, as the PTCI was not administered at follow-up timepoints. All reported models adjusted for age, sex, and cohort type (military sexual trauma vs. combat trauma). Likelihood ratio tests suggested that random intercepts and trend components led to greater model fit (*p*s < .001). Further, models assuming conditional independence of errors were found to be preferable to other autocorrelated error structures upon examination of Akaike Information Criterion (AIC) and Bayesian Information Criterion (BIC) values, and these fit indices as well as likelihood ratio tests supported random intercepts components, but not random slopes.

To test significant symptom worsening following programme completion, *t*-test and chi-square tests were used to examine relationships between age and sex, as well as symptom severity at baseline and post-treatment and changes in symptom severity over the course of treatment, symptom worsening, and missingness at the 3-month follow-up timepoint. For these analyses, symptom worsening was defined as a 10-point increase on the PCL-5 or a 3-point increase in the PHQ-9 from post-treatment to 3-month follow-up.^1^^,^^2^ A 3-point change on the PHQ-9 has previously been suggested to represent clinically meaningful change (Kroenke et al., 2016). A similar suggestion for clinically meaningful change on the PCL-5 does not currently exist, which is why we adopted the 10-point change that has been suggested for a previous version of the instrument (Weathers et al., 2013). Inferential analyses were conducted in Stata 14 (StataCorp, 2015) and Supermix 1.1 (Hedeker, Gibbons, Du Toit, & Cheng, 2008). Figures were created in Sigmaplot 13 (Sigmaplot, 2014).

## 3. Results

There were 192 (91.9%) veterans who completed the PCL-5 and PHQ-9 on the last day of the programme. Reasons for not completing final measures included the PCL-5 (past week version) not being added to programming until June 2016 and veterans having to leave the programme prior to being able to complete post-treatment assessments. Veterans who did or did not complete the PCL-5 and PHQ-9 on their last day in the programme did not statistically differ in age, sex, or their PCL-5 and PHQ-9 intake scores. Of the 209 veterans who started treatment, 118 (56.5%), 99 (47.4%), and 77 (36.8%) completed the PCL-5 and PHQ-9 as part of 3-, 6-, and 12-month follow-up assessments, respectively. At 3-, 6-, and 12-month follow-up timepoints, 114 (96.6%), 96 (97.0%), and 76 (98.7%) of those who completed the PCL-5 and PHQ-9 assessments answered questions about aftercare, respectively. The remaining veterans did not complete all assessments. Of the veterans who completed the aftercare survey, 102 (89.5%), 85 (88.5%), and 66 (86.8%) reported seeing a mental health provider at 3-, 6-, and 12-month follow-up, respectively. No relationships existed between missingness and amount of PCL-5 (*p* = .74), PHQ-9 (*p* = .52), or PTCI (*p* = .40) change from pre- to post-treatment. Additionally, no differences existed in age, sex, cohort type, or the proportion of clients meeting with a mental health provider during the follow-up period (*p*s > .20) between those who were and were not lost to follow-up.^3^

**Figure 1.**
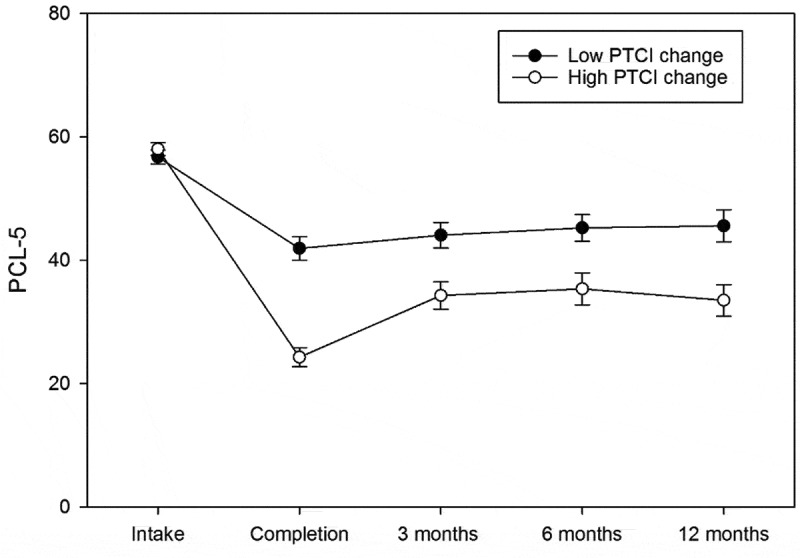
PTSD symptom change over time by high and low PTCI change.

**Figure 2.**
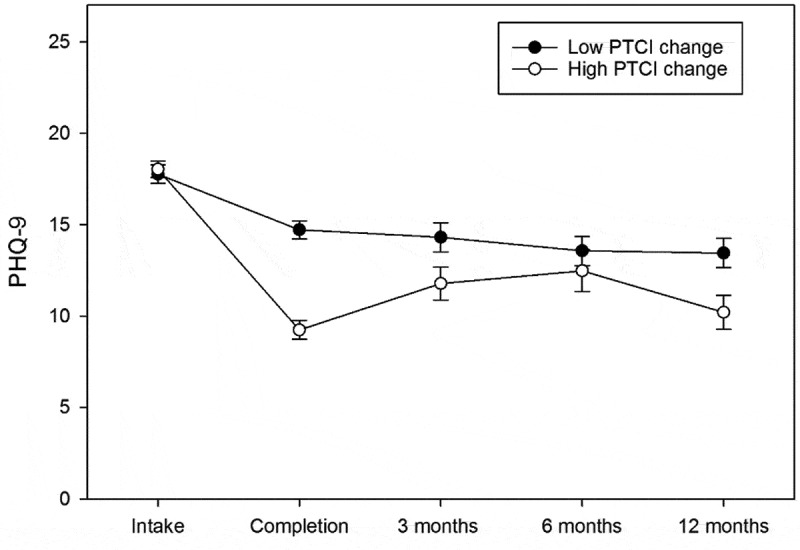
Depression symptom change over time by high and low PTCI change.

Overall, changes from pre-treatment to the 12-month follow-up timepoint were large for both the PCL-5 (*d* = 1.28, *p* < .001) and PHQ-9 (*d* = 1.18, *p* < .001). Results of random-effects longitudinal analysis indicated that quadratic and cubic time components existed for both the PCL-5 and PHQ-9 (*p*s < .001). These findings suggest that an initial reduction in outcome scores was followed by a slight symptom increase from post-treatment to the 3-month follow-up timepoint (see Table 2). Greater reductions in PTCI scores from pre- to post-treatment were associated with lower overall PCL-5 (*p* < .001) and PHQ-9 scores (*p* = .005) across time (see Figures 1 and 2, respectively). Changes in PTCI scores from pre- to post-treatment accounted for 12.22% of the variance in total PCL-5 change from intake to 12-month follow-up and 10.64% of the total change in PHQ-9 scores.^4^ Age, sex, and cohort type did not predict PCL-5 or PHQ-9 scores at any timepoint (*p*s > .20).

**Table 2.** Parameter estimates for PTSD and depression models.

Variable^a^	PCL-5 *b(SE)*	PHQ-9 *b(SE)*
Time	−17.11 (1.19)*	−4.50 (0.40)*
Time^2^	3.78 (0.30)*	0.99 (0.10)*
Time^3^	−0.20 (0.02)*	−0.05 (0.01)*
Age	−0.03 (0.09)	−0.02 (0.03)
Sex	−0.12 (2.59)	−0.17 (0.95)
Cohort Type	−3.04 (2.63)	−1.04 (0.97)
PTCI Change	−0.08 (0.02)*	−0.03 (0.01)*

PTCI Change refers to the pre-post difference in Posttraumatic Cognitions Inventory (PTCI) score.

^a^Month was used as the unit of time for time parameter estimates. Reference categories (coded 0) were male and military sexual trauma (MST) cohort.

* Variable is a significant predictor of outcome at *p* <.05.

PCL-5: PTSD Checklist for DSM-5. PTCI: Posttraumatic Cognitions Inventory. Median split for PTCI change was used to identify groups for those high or low in PTCI change during the programme for illustrative purposes. Error bars represent standard error.

PHQ-9: Patient Health Questionnaire-9. PTCI: Posttraumatic Cognitions Inventory. Median split for PTCI change was used to identify groups for those high or low in PTCI change during the programme for illustrative purposes. Error bars represent standard error.

Post-hoc comparisons of individual timepoints indicated that significant and meaningful reductions in both PCL-5 (*d* = 1.53, *p* < .001) and PHQ-9 (*d* = 1.09, *p* < .001) scores occurred from pre- to post-treatment. From post-treatment to the 3-month follow-up timepoint, small but statistically significant increases in PCL-5 (*d* = 0.30, *p* = .004) and PHQ scores (*d* = .16, *p* = .026) were observed. Following these slight symptom increases, no further significant changes occurred in either PCL-5 or PHQ-9 scores between 3- and 12-month follow-up timepoints (*p*s > .10; see Table 3).

**Table 3.** PCL and PHQ score distributions over time.

Outcome	Intake *(n = 209)* *M*(*SD*)	Programme Completion *(n = 192)^a^* *M*(*SD*)	3-month follow-up *(n = 118)* *M*(*SD*)	6-month follow-up *(n = 99)* *M*(*SD*)	12-month follow-up *(n = 77)* *M*(*SD*)
PCL-5	57.41 (11.39)	33.18 (19.20)	38.76 (17.40)	40.06 (17.49)	38.83 (17.09)
PHQ-9	17.90 (4.86)	11.89 (6.06)	12.94 (6.69)	13.00 (6.86)	11.64 (5.69)

PCL-5 refers to PTSD Checklist for DSM-5 total score, PHQ-9 refers to Patient Health Questionnaire-9 total score. ^a^Only includes veterans who completed the PCL-5 and PHQ-9 on the final day of the programme.

Veterans who experienced significant PTSD symptom worsening following treatment based on PCL-5 scores at the 3-month follow-up timepoint (*n* = 39, 36.79%) had lower PCL-5 scores at post-treatment (*d* = 1.06, *p* < .001), and greater PCL-5 change from pre- to post-treatment compared to those who did not experience a PTSD symptom worsening (*d* = 1.14, *p* < .001). Similarly, those who experienced significant depression symptom worsening following treatment based on PHQ-9 scores at the 3-month follow-up timepoint (*n* = 42, 37.84%) had lower post-treatment PHQ-9 scores (*d* = .72, *p* < .001) and greater PHQ-9 change from pre- to post-treatment (*d* = 0.80, *p* < .001). Change in PTCI scores from pre- to post-treatment was not related to symptom worsening based on PCL-5 (*d* = .37, *p* = .067) or PHQ-9 (*d* = .29, *p* = .137). Age, sex, and seeing a mental health provider during the follow-up period were also not significantly associated with maintenance of treatment gains (*p*s > .20).

## 4. Discussion

The current study evaluated long-term outcomes following a 3-week CPT-based ITP for PTSD to determine whether veterans tend to maintain treatment gains, experience symptom worsening, or continue to improve following the transition back to their home environment and into their local care system. Our results indicated that veterans maintained significant PTSD and depression symptom reductions made during the programme for up to 12 months following programme completion with a minimal increase in symptoms at 3-months followed by symptom stabilization. The observed pre-treatment to 12-month follow-up effect sizes are consistent with the longer-term follow-up literature for traditional treatment delivery formats (Resick et al., 2012), providing further support for the effectiveness of intensive PTSD treatment programmes.

Notably, the majority of veterans in the present study (over 85%) followed aftercare recommendations and reported seeing a mental health provider at the follow-up timepoints. Continuing to receive general mental health support following ITP completion is a relatively common practice associated with intensively-delivered cognitive behavioural treatments (Held et al., 2019a). It is possible that aftercare may have played an important role in preventing symptom worsening. Seeing a mental health provider at the follow-up timepoints was not a significant predictor of symptom maintenance, suggesting that the 3-week ITP can help some individuals maintain their gains in the longer term even if they do not continue receive mental health care upon treatment completion. However, this analysis could have been underpowered due to uneven groups. Moreover, it is possible that the individuals who were more likely to have symptom worsening were also more likely to seek out aftercare. Given that, on average, veterans in the present sample continued to report symptoms above the cut-off for probable PTSD (PCL-5 > 33; Bovin et al., 2016) at post-treatment and at all follow-up time points, further research is needed to identify individuals who may or may not require additional mental health treatment following intensive PTSD treatment and determine what types and frequency or dose of aftercare could help to promote further gains to remission following intensive treatment programs.

Our study found that veterans who experienced greater reductions in negative posttrauma cognitions from pre- to post-treatment reported significantly lower PTSD and depression symptoms during the follow-up period. These findings support one of the key mechanisms associated with symptom change in trauma-focused therapies (Zalta, 2015) and extend previous research demonstrating that changes in negative posttrauma cognitions are an important predictor of PTSD symptom reduction over the course of intensive PTSD treatment (Zalta et al., 2018). Moreover, these findings are consistent with studies that have identified changes in negative posttrauma cognitions over the course of traditionally-delivered CPT as key predictors of longer-term outcomes (Holliday, Link-Malcolm, Morris, & Surís, 2014; Owens, Pike, & Chard, 2001; Scher, Suvak, & Resick, 2017) and highlight the importance for clinicians to target negative posttrauma cognitions to increase the chance for maintenance of treatment gains achieved over short treatment periods. However, changes in negative posttrauma cognitions may only be one of the important mechanisms. Recent research has suggested that yoga and mindfulness interventions may have some benefits for individuals with PTSD (Davis et al., 2019; Nguyen-Feng, Clark, & Butler, 2019). Thus, future studies examining intensive PTSD treatments that combine evidence-based PTSD treatments with integrative interventions (e.g., mindfulness and yoga) should also evaluate the mechanisms by which these interventions may contribute to initial symptom reduction and longer-term maintenance of treatment gains.

Following the ITP completion, veterans reported a relatively small but statistically significant increase in PTSD symptom severity before remaining stable for the remainder of the one year follow-up period. One explanation for the small increase in PTSD symptoms could be the transition from the relatively structured ITP to less structured home environments with additional stressors. The relatively small increase in symptoms and the general maintenance of gains long-term suggests that veterans appear to be able to apply the skills they acquired during the ITP following treatment. These findings further support the feasibility and effectiveness of delivering intensive trauma-focused evidence-based therapies (Held et al., 2019b).

Slightly more than one third of veterans were unable to maintain their treatment gains in PTSD or depression symptoms following the completion of the ITP. Surprisingly, the veterans who experienced symptom worsening had significantly lower post-treatment symptom scores and reported significantly greater changes in PTSD and depression symptoms over the course of the programme. No measured variables were predictive of PTSD and depression symptom worsening following ITP completion. It is possible that the ITP structure led veterans to feel as though they had made greater improvements than they may have actually made, which may have led them to rate their post-treatment symptoms as minimal. Returning into their home environments may have led them to realize that some of their symptoms were unresolved or may have simply not occurred during the ITP.

This study had several limitations. Aside from the structured diagnostic assessments during the intake to determine fit for the ITP, all measures were self-report. Self-reported symptoms may not accurately reflect clinical diagnoses, especially given the documented tendency for military personnel to underreport symptoms (Hoge et al., 2004). It is also possible that some of the perceived improvements following the ITP may be the results of reporting biases. Given the lack of a control group of veterans who did not continue to work with mental health providers following the ITP, we are unable to determine whether individuals would have maintained their treatment gains without the continued support. Relatedly, we did not inquire about negative posttrauma cognitions, continued utilization of CPT, or utilization of other skills acquired during the ITP at follow-up timepoints. We are therefore unable to determine whether the maintenance of gains is directly associated with the ITP completion. Due to sample size and power limitations, we were unable to explore a larger range of predictors of symptom worsening following the ITP. It is plausible that several factors that we were not able to examine in this study, such as the veterans’ continued practice of skills gained (e.g., CPT skills, mindfulness and yoga practice), substance use following the completion of the ITP, and the type and dose of aftercare they received, may also be important predictors of symptom worsening after completing the ITP. Although follow-up survey completion rates were acceptable for clinical research, only a relatively small number of individuals completed the assessments at the 12-month follow-up timepoint. It is possible that results could have differed had all veterans responded to the follow-up surveys. Specifically, since it is unlikely that data were missing completely at random, the currently large effect size from pre-treatment to 12-month follow-up may be an overestimate. Lastly, given the design of the clinical programme, we were unable to determine which of the interventions offered during the ITP contributed the most to treatment success. Although CPT is considered a core component of the ITP, recent research has shown that mindfulness and yoga practice can also significantly contribute to PTSD symptom reductions (Davis et al., 2019; Nguyen-Feng et al., 2019). Thus, any findings need to be attributed to the ITP in general rather than any of the specific programme components.

Despite these limitations, this study supports the longer-term effectiveness of a CPT-based ITP for veterans with PTSD. Large clinical gains can be made when treatment is delivered daily over the course of only 3 weeks and maintained for up to 12 months. Moreover, this study extends prior research by demonstrating that reductions in negative posttrauma cognitions over the course of the ITP predict longer-term symptom maintenance. Lastly, our findings suggest that several factors, such as lower self-reported PTSD and depression symptom severity at endpoint and greater self-reported PTSD and depression symptom change over the course of the programme predicted worsening in the respective symptoms three months following the ITP. Overall, the present study provides additional support for the notion that intensively-delivered PTSD treatments show great promise (Held et al., 2019a). Randomizing individuals into receiving and not receiving follow-up care can further clarify the role of aftercare on maintenance of symptom gains. Future research should evaluate strategies that can facilitate the maintenance of reduced PTSD symptoms and factors that may affect symptom worsening following the completion of the ITP.

## Data Availability

Datasets generated and analyzed during the current study are not publicly available because they contain more than two indirect identifiers of human research participants that cannot be sufficiently anonymized for a public repository. The datasets are available from the corresponding author on reasonable request (Held, 2019).

## Notes

1. We decided on this definition of symptom worsening rather than applying Beidel et al. (2017) definition of relapse because clinician-administered PTSD measures were not utilized other than during intake and because we conceptualized symptom worsening not only as a return to baseline functioning but a general inability to maintain a symptom gain. For example, individuals may experience symptom worsening following treatment but still have much improved symptom severity ratings compared to their baseline functioning.

2. Due to arbitrary cut-offs used here failing to account for individual differences in symptom change and fluctuations during treatment, we also examined relapse defined as a one-standard deviation increase in outcome scores based on each individual’s standard deviation of repeated symptom measurements during the 3-week ITP. The pattern of results was the same as reported here.

3. As a sensitivity analysis to ensure bias in attrition did not affect results of post-hoc comparisons, timepoint comparisons were also conducted using listwise deletion, which yielded the same significance pattern of results.

4. Reported *R*^2^ estimates are based on shared variance between outcome total change scores and programmatic PTCI change scores accounting for age, sex, and cohort type, but do not account for random effects. Using Nakagawa and Cuthill (2007) random effects-based procedure, estimates of effect size for PTCI change were *r* =.15 for PCL and *r* =.13 for PHQ in the reported adjusted models.
